# 4223 Changes in positive predictive value of cervical cytology following HPV vaccination

**DOI:** 10.1017/cts.2020.113

**Published:** 2020-07-29

**Authors:** Deanna Teoh, Gwiwon Nam, Shalini Kulasingam, Rachel I. Vogel

**Affiliations:** 1 University of Minnesota CTSI; 2 University of Minnesota

## Abstract

OBJECTIVES/GOALS: To determine if current U.S. HPV vaccination rates have decreased dysplasia prevalence enough to decrease the positive predictive value (PPV) of abnormal cervical cytology. METHODS/STUDY POPULATION: This retrospective cohort study comprised a chart review of all patients 21-35 years of age who had at least 1 Pap test result within MHealth/Fairview 2016-2018. HPV vaccination data, cervical cancer screening data and dysplasia results were abstracted. Vaccinated was defined as receiving at least 1 dose of HPV vaccine, with subgroup analyses performed for those completing vaccination per ACIP guidelines and by age of initiation dichotomized as 21+ years versus <21 years. RESULTS/ANTICIPATED RESULTS: 49,764 patients meeting study criteria were identified. Among the entire study population, 10% had abnormal cytology results during the study period. Among the 4,928 patients with abnormal cytology, PPV for CIN2+ was lower among vaccinated individuals (13% vs. 18%; p < 0.0001). Among vaccinated individuals, PPV was lower among those completing vaccination (12% vs. 16% for incomplete vaccination; p = 0.04) and among those initiating vaccination at <21 years of age (9% vs 26% for 21+y; p < 0.0001). DISCUSSION/SIGNIFICANCE OF IMPACT: Among a population with low HPV vaccine coverage, the decrease in dysplasia prevalence among vaccinated individuals is resulting in a subsequent decrease in PPV of cervical cytology, particularly in those initiating vaccination prior to 21 years of age and among those completing the series. Confirmation of these results will call for changes in screening strategies for vaccinated individuals. CONFLICT OF INTEREST DESCRIPTION: Acelity: Industry grant for an investigator-initiated industry-sponsored clinical trial. Tesaro: Site PI for industry-sponsored clinical trial. NOTE: Funding from the industries above are unrelated to the research presented in the abstract.

